# From Molecules to Materials, Devices and Processes: The Chemical Basis of Novel Technologies

**DOI:** 10.3390/molecules30020357

**Published:** 2025-01-17

**Authors:** Giuseppina Raffaini, Fabio Ganazzoli

**Affiliations:** 1 Department of Chemistry, Materials and Chemical Engineering “G. Natta”, Politecnico di Milano, Via Luigi Mancinelli 7, 20131 Milan, Italy; 2 INSTM, National Consortium of Materials Science and Technology, Local Unit Politecnico di Milano, 20131 Milano, Italy

## 1. Introduction

This Special Issue was launched in connection with the joint XIII National Congress of AICIng (the Italian Association of Chemistry for Engineering) and the II National Congress of the Division of Chemistry for the Technologies of the Italian Chemical Society, held at the Politecnico di Milano (Italy) from 25 to 28 June 2023. The aim of the Congress was to present and discuss recent advances in fundamental and applied chemistry in the fields of new or improved technologies, comprising compounds, materials or processes.

## 2. Scope of the Special Issue

On the Special Issue’s web page (https://www.mdpi.com/journal/molecules/special_issues/O8G0TVX1RU last accessed on 16 January 2025), we pointed out that chemistry provides a molecular-level tool, allowing for significant advances in current and novel technologies in a wide range of fields. In this Special Issue, we collected selected relevant examples of the chemical foundations of the technologies that produced the new, scientific, and technologically innovative results obtained in Italy and elsewhere, in which various methodologies and approaches were used to design new products, materials, or processes in a wide range of fields.

It must be noted that there is a common thread to the papers collected in the present Special Issue, which is the sustainability of the products, materials and processes, as seen from a chemical viewpoint, and their novel technological applications. Sustainability is considered here in different ways, ranging from green chemistry (in a strict sense) to the circular economy (especially the waste-to-product processes), as well as the performance of the modified materials, including composites such as tires in the automotive sector or the eco-sustainable synthesis of nanomaterials for energy. Other sustainability issues encountered in agriculture include the food traceability of crops, including the detection of possible fraud or industrial processing and the preparation of biocompatible and biodegradable natural pesticides to allow for sustainable crop production with a minimal or negligible environmental impact. In addition, new materials with improved performances, processes, or materials related to renewable energy or to energy storage are presented, and the increasingly important topic of environmental remediation is discussed.

## 3. Overview of the Papers in the Special Issue

Green chemistry [1,2,3] is a major consideration in the design of products, materials, or processes that aim to minimize or eliminate unwanted or possibly hazardous byproducts, improving their atom economy as much as possible. In this way, both the environmental impact and the use of non-renewable resources are minimized or completely avoided [4]. A fitting example of this approach is provided by Naddeo et al. (Contribution 1), who carried out a one-pot synthesis of 2,5-hexanedione (HD) via a ring-opening reaction of 2,5-dimethylfuran (DF), obtaining a very high yield. The HD was then used for the synthesis of pyrrole derivatives with various amines, again obtaining a very high yield, with water as the only byproduct. This process had a very high carbon efficiency and a very small E-factor, shown as the ratio of the waste mass to the product mass, which is a remarkable result. It should be added that, in the same paper, one of the pyrrole derivatives was used to functionalize the carbon black used as a filler in an elastomeric composite; this functionalized carbon black showed enhanced mechanical properties, which could improve the sustainability of tires in the automotive sectors [5,6], and, as such, is presently undergoing industrial-scale development. A somewhat related paper (Contribution 2) carried out a theoretical study of the adsorption of linear and cyclic molecules, both saturated and unsaturated, on a pristine graphene surface using DFT methods after a first study based on the molecular mechanics and molecular dynamics of the adsorption process [7]. The adsorption energies and the charge difference density were used to understand the intermolecular interactions at play. The adsorption energy was found to be dependent on the number of π electrons, with the most favorable results being provided by pyrrole-oxidized derivatives, providing a quantitative measure of the stability of the surface modifications offered by the physisorption of small molecules. This procedure allows for the production of novel fillers that could improve the mechanical properties of polymer composites [8] such as those used in tires.

Quantitative assessments of the environmental impact of a model organic synthesis, such as that carried out by Ruini et al. (Contribution 3), are strongly related to the green chemistry philosophy. This study achieved process optimization through a multivariate experimental design, aiming to maximize the yield of the desired product and, at the same time, to minimize the environmental impact, as quantified using the life cycle assessment (LCA) methodology [9,10]. The selected reaction was a nucleophilic substitution of a phenolic compound, namely vanillin, which was obtained from a renewable source, lignin. The approach presented in this paper, using a combination of an experimental design and a life cycle assessment, appears to be a step forward in measuring and improving the sustainability of organic synthesis processes.

Another type of organic synthesis was investigated in Contribution 4, in which different bispidine derivatives were prepared. Bispidine is a bicyclic diamine that may act as a ligand towards metal cations, particularly when additional nitrogen atoms are introduced via derivatization, with potential applications in many fields. In this paper, triazole derivatives of bispidine were prepared using the click reaction, which provided a high yield, particularly when performed under microwave irradiation. Note that this procedure appears to be in line with the principles of atomic efficiency, which is a common issue in green chemistry. Furthermore, the zinc(II) and copper(II) complexes of some of the obtained bispidine were also shown to be efficient catalysts for the Henry reaction, which is an established C-C bond-formation reaction. Once again, excellent yields were obtained under microwave irradiation, which appears to be a useful tool in many reactions to enhance the product yield.

A different methodology was employed to study the organic synthesis of bis(indolyl)methanes (Contribution 5), which are natural bis-heterocyclic compounds that are important organic intermediates in the pharmaceutical and chemical industries. The unique approach adopted in this paper is the proposed use of magnetic aerogels as catalysts, exploiting their high and open porosity and tunability and their ability to stabilize active metal-based nanocrystals. The investigated catalysts were highly porous NiFe_2_O_4_/SiO_2_ aerogel nanocomposites containing ferrite nanocrystals with a size of about 10 nm. These aerogels were acidic catalysts that provided an excellent yield of bis(indolyl)methane when starting from stoichiometric amounts of indole and 4-nitro-benzaldehyde.

A different aspect of the sustainability of products or processes is the circular economy [11], which focuses on the means of recycling or reusing existing materials and products as much as possible, thus positively affecting environmental use and waste management. Several papers in this Special Issue provide examples of the circular economy. One review paper (Contribution 6) provided an overview of the current state of the art in the field of depolymerization or the degradation of polypropylene. For this polymer, the relevant technologies focus on advanced oxidation. In fact, the thermal recycling of this thermoplastic polymer was carried out for only a fraction of the discarded material, with the best choice being the back-transformation of polypropylene in the starting monomer, which can be purified and then repolymerized. However, these technologies are also of great importance in the removal of microplastics from water and wastewater [12,13,14], where they are found as pollutants originating from domestic and personal care products, with a direct environmental impact.

Among the other papers that deal with the circular economy, the study carried out by Atanasio et al. (Contribution 7) investigated the one-pot synthesis of graphene quantum dots from carbon aerogels obtained from rice husk, an abundant form of agricultural waste. Interestingly, the cellulose derived from the rice husk after gelification and carbonization was shown to produce quantum dots without the use of any solvent via ball-milling, obtaining the goals of the green chemistry approach. In turn, the quantum dots were then used as electrode materials for supercapacitors and Li-ion batteries, showing their usefulness as nanomaterials in advanced systems of energy storage [15,16,17].

Another paper (Contribution 8) studied a different waste of agricultural origin, namely soybean whey wastewater, in order to enrich soybean trypsin inhibitors. This procedure may have the twofold effect of reducing the environmental pollution of soybean whey and of recovering trypsin inhibitors, which have important physiological functions in the health food and pharmaceutical fields.

Concerning different types of waste, another paper (Contribution 9) considered the possibility of recycling a fast-growing type of e-waste (i.e., electronic waste) consisting of waste printed circuit boards. The glassy substrate of the non-metal fraction of the electronic boards was treated with acidic leaching with the help of microwave heating and was shown to be an excellent adsorbent against methylene blue, matching the performance of activated carbon. In view of the ever-increasing production of e-waste, this work offers the interesting possibility of recycling the printed boards after removing the metals, such as copper, for instance, and noble metals such as gold. Another contribution (Contribution 10) addressed the issue of the separation of Co(II) from Ni(II) in extractive metallurgy, as well as from spent Li-ion batteries, where these metals are present in the cathode; hence, this paper may also be relevant to the circular economy. The proposed method explores the use of a room-temperature ionic liquid via liquid–liquid extraction in a strongly acidic medium and through the polymer inclusion membranes formed of PVC and the same ionic liquid. The latter procedure was found to be the best one, since it used a lower amount of ionic liquid and allowed for the efficient separation of the metal cations in a single stage [18].

Other approaches investigated the issue of food control and the traceability of foodstuffs, particularly tomato samples (Contribution 11). In this case, using NMR spectra obtained in different laboratories via spectroscopic fingerprint data, one could assess the geographical origin of the tomatoes, as well as possible fraud, production, and industrial processing with high reliability using a metabolomic analysis conducted via a statistical multivariate data analysis. Another paper (Contribution 12) dealt with the issue of sustainability in agriculture through the preparation of pesticides based on essential oils stabilized in an appropriate oil-in-water emulsion. The essential oils were obtained through supercritical CO_2_ extraction (in keeping with the requirements of green chemistry) and were encapsulated in stabilizing cellulose nanocrystals crosslinked with calcium chloride into an optimized ratio. These sustainable eco-friendly systems were active biopesticides in both in vitro and in vivo tests on olive seedlings, with the additional advantage of the use of a biocompatible and biodegradable nanocellulose stabilizer.

Finally, we should mention one approach to sustainability from a somewhat different perspective, namely the topic of renewable energy and environmental remediation. The separation of CO_2_ from the biogas obtained from the anaerobic digestion of various organic wastes is important to obtain biomethane, which is useful in gas grids. One paper in this Special Issue (Contribution 13) tackled this problem by using a commercial metal–organic framework (MOF) extruded with a polymer in order to increase its stability, finding that hydrophobic polymers such as polyurethane were the best choice in view of the water sensitivity of the MOF. As a result, the CO_2_ separation was also more complete than that obtained with the isolated MOF. In a different context, the removal and sequestration of CO_2_ from the atmosphere due to industrial exhausts were investigated in another paper (Contribution 14). Here, the disposal of CO_2_ in a geological formation was not considered; instead, the contribution focused on the possibility of storing it in seawater in the form of bicarbonate ions, avoiding the formation of calcium carbonate, which would release one half of the previously disposed CO_2_. The limits of CO_2_ storage in seawater at its natural pH and salinity (using the Mediterranean Sea as an example) were thus investigated both in the laboratory and in a pilot plant.

Finally, a more fundamental study (Contribution 15) addressed the problem of sol–gel transition, particularly the onset of gelation in polymer solutions, comparing a new theoretical model with the onset of gelation in agar probed via viscosimetric experiments. This is a technologically relevant problem in the preparation of hydrogels, for instance, which are important in the food sector and in the cosmetic and pharmaceutical industries, among others, as well as in the preparation of 3D scaffolds such as those used in tissue engineering [19].

## 4. Conclusions

In conclusion, we believe that the papers collected in this Special Issue provide an interesting overview of the current chemical approaches to novel technologies in terms of compounds, materials, and processes that are being carried out in Italy and elsewhere. The common thread is sustainability, which is considered from different viewpoints across a wide spectrum of problems and methodologies.
